# Microstructure Evolution and Solute Segregation of Inconel 718 in Laser Additive Manufacturing: A Numerical and Experimental Investigation

**DOI:** 10.3390/ma19081642

**Published:** 2026-04-20

**Authors:** Hang Liu, Wenjia Xiao, Baolin Yan, Hui Xiao

**Affiliations:** 1 Guangdong Provincial Key Laboratory of Industrial Intelligent Inspection Technology, Foshan University, Foshan 528001, China; liuhangfosu@126.com (H.L.); 418m@163.com (B.Y.); xiaohui1113@fosu.edu.cn (H.X.); 2 School of Materials Science and Engineering, Central South University, Changsha 410083, China

**Keywords:** nickel-based superalloys, laser additive manufacturing, scanning speed, solidification parameters, microstructural evolution

## Abstract

The segregation of brittle Laves phases remains a critical bottleneck limiting the performance of additive manufacturing (AM) nickel-based superalloys. While its evolution is governed by complex transient physical fields within the melt pool, a quantitative kinetic correlation between processing parameters and microstructural features is currently lacking. In this study, a high-fidelity multiphysics numerical model was developed to establish a cross-scale mapping logic of “Process-Physical Field-Microstructure” by dissecting the global distribution of temperature gradient (*G*) and solidification rate (*R*) along the quasi-steady-state melt pool boundary. It is revealed that increasing the scanning speed synergistically enhances *R* while compressing *G*. Beyond driving a transition from oriented columnar dendrites to refined mixed-dendritic structures, this shift effectively blocks the continuous enrichment channels of Nb and Mo elements by compressing the “kinetic time window” for solute redistribution. Consequently, the morphology of the Laves phase is forced to evolve from a continuous interconnected chain-like network into dispersed isolated particles. This research clarifies the kinetic essence of microstructural evolution under non-equilibrium solidification, providing critical physical criteria for the precise intervention of deleterious phases and the regulation of microstructural consistency in high-performance AM components.

## 1. Introduction

Additive manufacturing (AM), characterized by its layer-by-layer deposition capability and flexible forming process, has enabled breakthrough advancements in the fabrication of complex three-dimensional structures with high material utilization efficiency. Consequently, it has become a key manufacturing technology in strategic fields such as aerospace and energy equipment [1,2,3]. Among the various material systems applicable to AM, nickel-based superalloys are widely regarded as essential materials for critical components in aero-engine hot sections and gas turbines due to their outstanding high-temperature strength, creep resistance, and resistance to oxidation and corrosion [4,5]. Laser additive manufacturing (LAM) not only significantly improves the manufacturing efficiency of these complex components but also offers unprecedented potential for lightweight structural design and functional integration that are difficult to achieve using conventional manufacturing processes [6,7].

However, the extreme thermo-physical conditions inherent in the LAM process impart pronounced non-equilibrium solidification characteristics to nickel-based superalloys [7,8]. Under conditions of high temperature gradients and extremely rapid cooling rates, severe elemental segregation inevitably occurs within the alloy. In particular, solute elements such as Nb and Mo tend to accumulate in interdendritic regions, directly leading to the precipitation of brittle Laves phases [9,10]. Previous studies have demonstrated that these chain-like distributed secondary phases not only deteriorate the mechanical properties and service reliability of the material but also significantly suppress the precipitation kinetics of subsequent strengthening phases. Lim et al. [11] reported that rapid solidification and repeated thermal cycling during additive manufacturing typically lead to the formation of pronounced dendritic structures in Inconel 718 alloys, accompanied by Nb segregation in interdendritic regions and the precipitation of Nb-rich Laves phases, which adversely affect material performance and subsequent precipitation behavior of strengthening phases. Similarly, Bassini et al. [12] found that during laser powder bed fusion, the limited diffusion capability of Nb promotes its enrichment in interdendritic regions, facilitating the formation of brittle Laves phases and consequently degrading mechanical properties. Kong et al. [13] further indicated that the enrichment of Nb and other solute elements in the interdendritic liquid during rapid solidification is a primary driving factor for Laves phase formation, thereby promoting the precipitation of interdendritic secondary phases. Although previous studies have attempted to optimize the microstructure by adjusting process parameters such as laser power and scanning speed, the highly transient and multiphysics-coupled thermal–fluid–solidification behavior within the melt pool makes the microstructural response extremely complex and uncertain.

Fundamentally, the temperature gradient *G* within the melt pool and the solid–liquid solidification rate *R* serve as the key physical criteria governing crystal growth modes and solute redistribution behavior during solidification. Heo et al. [14] reported that in laser additive manufacturing of Inconel 718 alloys, key process parameters such as laser power and scanning speed significantly influence the thermal history and solidification behavior of the melt pool, thereby determining the resulting microstructural characteristics and mechanical properties. In addition, Ghorbanpour et al. [15] demonstrated that variations in scanning speed and laser power can alter the melt pool temperature field and cooling rate, leading to different microstructural morphologies. Nevertheless, a critical scientific challenge remains: conventional experimental techniques are limited in their ability to perform in situ measurements of the transient temperature field and solidification parameter distributions within the melt pool due to the extremely small spatial scale and rapid dynamic evolution involved. Schneider et al. [16] noted that the repeated rapid melting and solidification during additive manufacturing generate highly non-equilibrium temperature fields and solidification conditions, which directly influence elemental segregation behavior and microstructural evolution. As a result, most previous studies have been largely confined to empirical correlations between processing parameters and microstructural features, while the dynamic governing role of the coupled evolution of *G* and *R* has often been overlooked. Wang et al. [17] further emphasized that it is difficult to directly measure the transient temperature field and microscopic solidification behavior within the melt pool through experimental approaches alone, highlighting the necessity of numerical simulations to investigate the internal microstructural evolution process. The lack of quantitative analysis of global interfacial solidification parameters has long hindered the precise control of harmful phase formation and microstructural consistency in additive manufacturing, leading to a reliance on trial-and-error approaches for process optimization [18,19].

To address these challenges, this study overcomes the limitations of conventional experimental observation by developing a high-fidelity multiphysics numerical model aimed at fundamentally elucidating the mechanisms governing microstructural evolution during continuous-wave laser additive manufacturing (CW-LAM). By quantitatively extracting key solidification parameters across the entire liquidus boundary of the melt pool, including *G*, *R*, and their combined parameters, the present work systematically clarifies the regulatory effect of scanning speed on melt pool solidification behavior. Furthermore, combined with microstructural characterization, this study reveals the physical mechanism by which the formation of Laves phases can be suppressed through the compression of the kinetic time window for solute redistribution. The findings not only provide deeper insights into the microstructural formation mechanisms of nickel-based superalloys but also offer a deterministic scientific basis for optimizing additive manufacturing process parameters and achieving coordinated control of microstructure and performance from a solidification kinetics perspective.

## 2. Experimental Design

### 2.1. Simulation Scheme

In the numerical simulation design, typical process parameters for laser cladding were selected to ensure engineering representativeness while maintaining clarity for parameter comparisons. It should be noted that the experimental fabrication and numerical simulations were conducted under the same processing conditions to ensure direct comparability between simulated results and observed microstructures. The laser power was set to 600W to provide a stable and sufficient energy input. Scanning speeds of 6mm/s and 10mm/s were chosen to investigate the differences in melt pool thermal–flow–solidification behavior under varying linear energy densities. The laser spot radius was set to 0.6mm, reflecting the commonly used spot size in practical processes. For the powder delivery process, a powder feed rate of 12g/min was employed, with a carrier gas flow rate of 10L/min to ensure continuous and stable powder transport.

Material properties were assigned based on the Inconel 718 (Table 1), including density $\rho \approx 8.19\times 10^{3}\;\mathrm{kg}/m^{3}$, thermal conductivity k≈11.4W/(m·K), and specific heat capacity $c_{p}\approx 435\;J/\left(\mathrm{kg}\cdot K\right)$ at room temperature, as widely reported in the literature. For high-temperature conditions approaching the melt, temperature-dependent properties were adopted to capture the material behavior more accurately: the density decreases slightly to ∼$7.9\times 10^{3}\;\mathrm{kg}/m^{3}$, the specific heat rises to ∼500–550J/(kg·K), and the thermal conductivity varies from ∼15–25W/(m·K) in the liquid state, while the solidus and liquidus temperatures are $T_{s}\approx 1528\;K$ and $T_{l}\approx 1609\;K$, respectively. By combining the above parameters, consistent experimental and simulation conditions were established to systematically analyze the effects of scanning speed on melt pool solidification behavior and subsequent microstructural evolution.

### 2.2. Geometrical Model and Mesh Generation

**Figure 1:**
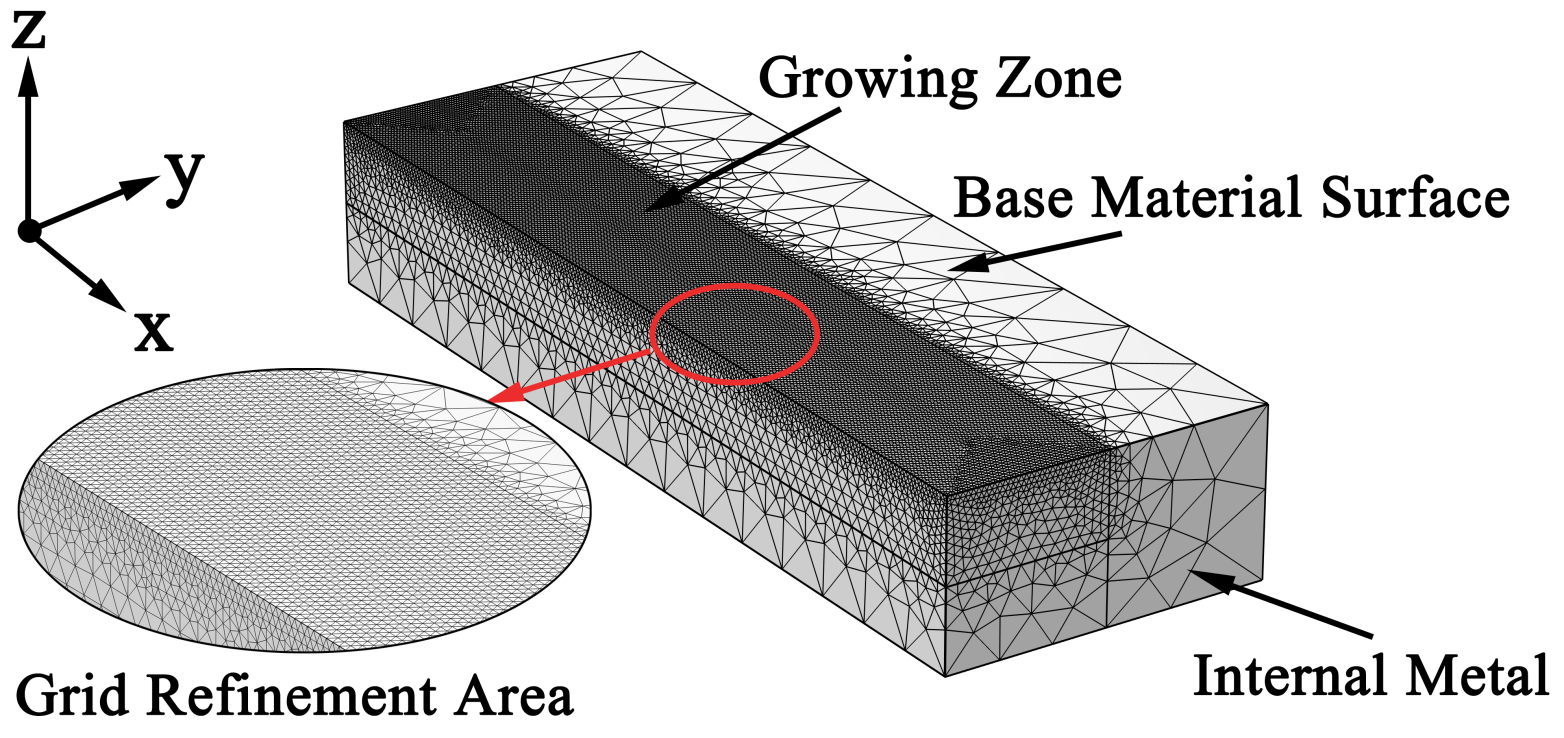
Geometrical model and mesh distribution.

The temperature field during laser cladding was calculated using the finite element method (FEM), enabling accurate capture and visualization of temperature data. To precisely resolve temperature variations within the clad layer, the mesh was refined along the laser scanning path on the substrate surface and in the region of clad layer growth. In contrast, regions where metal melting did not occur were assigned a coarser mesh. The final computational domain of the substrate consisted of 297,364 elements, and grid independence was confirmed through verification studies (Figure 1).

### 2.3. Laser Heat Source and Powder Layer Motion Model

The laser heat source was modeled using the Gaussian heat source, which is commonly employed in temperature field simulations. The laser beam intensity [18] was defined as

(1) $$Q\left(r\right)=\frac{2\eta P}{\pi R^{2}}\exp\left(-\frac{2r^{2}}{R^{2}}\right),$$

where *P* denotes the laser power, η is the material absorption efficiency of the laser energy, *r* is the radial distance from an arbitrary point to the center of the heat source, and *R* represents the effective radius of the heat source.

In the experiment, both the laser and the coaxial powder feeder move at a constant velocity *v* along the positive *x*-direction, starting from the substrate position at x=0mm. The corresponding expression is

(2) $$r^{2}={\left(x-v\;t\right)}^{2}+y^{2},$$

Here, *x* and *y* represent the Cartesian coordinates of each computational cell on the substrate, which are automatically provided by the simulation software. The variable *v* denotes the laser scanning velocity, and *t* is the time variable that varies as the simulation progresses.

The powder flow control was incorporated based on the laser heat source model by introducing a powder density term, enabling comprehensive control of the interface velocity of powder addition [16]. The corresponding expression is

(3) $$Q\left(r\right)=\frac{2\;\eta \;P}{\rho \;\pi \;R^{2}}\exp\;\left(-\frac{2r^{2}}{R^{2}}\right),$$

The introduction of the powder density term scales the total input per unit time by a factor of 1/ρ, while the overall powder distribution range remains unchanged, resulting in a reduced overall intensity of powder input.

### 2.4. Physical Field Parameters

The realistic morphological evolution of the clad layer in the simulation is governed by the combined effects of the Marangoni effect and volumetric fluid forces. The Marangoni effect controls variations in the liquid surface tension along the melt-pool free surface [11]. Temperature gradients along the free surface lead to surface tension gradients, which in turn generate tangential shear stresses that drive fluid flow within the molten pool.

For a three-dimensional melt pool, the Marangoni effect acts only along the tangential directions of the free surface. Therefore, the momentum boundary condition at the liquid–gas interface is expressed in terms of tangential stress balance. Since the free surface has two independent tangential directions, namely the *y*- and *z*-directions, the Marangoni boundary condition consists of two equations corresponding to these directions. The normal component of the stress is governed separately by the pressure and surface curvature balance and is therefore not included in the Marangoni shear condition. The tangential momentum boundary conditions can be written as

(4) $$\mu \;\frac{\partial v}{\partial x}=-\;\frac{\partial T}{\partial y}\;\left(d\gamma /dT\right),$$

(5) $$\mu \;\frac{\partial w}{\partial x}=-\;\frac{\partial T}{\partial z}\;\left(d\gamma /dT\right),$$

Here, μ is the dynamic viscosity of the liquid metal, γ is the surface tension coefficient, and *v* and *w* are the velocity components in the *y*- and *z*-directions, respectively. The terms ∂T/∂y and ∂T/∂z represent the temperature gradients along the free surface in the corresponding directions. The coefficient dγ/dT denotes the temperature dependence of surface tension, which characterizes the thermocapillary effect.

For the experimental material used in this study, Inconel 718, the temperature coefficient of surface tension dγ/dT is commonly treated as a constant with a value of approximately $-3.7\times 10^{-4}\;N\;m^{-1}\;K^{-1}$, according to reported thermophysical property measurements of molten Inconel 718. The surface tension at each node on the melt-pool surface is therefore determined by the product of this coefficient and the local temperature gradient along the free surface.

The volumetric fluid force is a non-contact force applied to the fluid by the external force field, whose magnitude is proportional to the mass or volume of the fluid. The corresponding [18] expression is

(6) $$\rho \;\frac{\partial u}{\partial t}+\rho \;\left(u\cdot \nabla \right)u=\nabla \cdot \left[-p\;I+\mu \;\left(\nabla u+{\left(\nabla u\right)}^{T}\right)-\left(\frac{2\mu }{3}-\kappa \right)\left(\nabla \cdot u\right)I\right]+F,$$

(7) $$\frac{\partial \rho }{\partial t}+\nabla \cdot \left(\rho \;u\right)=0.$$

Here, u is the fluid velocity, *p* is the fluid pressure, ρ is the material density, μ is the dynamic viscosity of the fluid, and κ is the bulk viscosity (assumed to be zero in this study). The source term F corresponds to a mushy-zone damping model, which is used to simulate the damping effect at the phase-change interface. This model introduces a momentum sink term in the mushy region that progressively dampens the fluid velocity as the material solidifies, allowing the velocity to approach zero in the solid phase and to vanish in the liquid phase. Such modeling is widely adopted in enthalpy-porosity and Darcy-type approaches to phase-change simulations, where a damping coefficient derived from the Carman–Kozeny equation is used to control the velocity reduction in the mushy zone [3,16]. The expression is

(8) $$F=-\;\frac{{\left(1-\gamma \right)}^{2}}{\gamma^{3}+\varepsilon }\;A_{\mathrm{mush}}\;\left(u-u_{\mathrm{cast}}\right),$$

In this expression, γ denotes the liquid volume fraction, and $A_{\mathrm{mush}}$ and ε are empirical constants, where $A_{\mathrm{mush}}$ is chosen to be sufficiently large and ε is taken to be sufficiently small to achieve an appropriate magnitude of damping. The parameter $u_{\mathrm{cast}}$ represents the reference casting velocity, and the difference between u and $u_{\mathrm{cast}}$ gives the relative velocity of the fluid with respect to the casting rod. In the mushy zone, when γ→1 (fully liquid), the damping term approaches zero, allowing fluid motion, whereas as γ→0 (fully solid), the damping term becomes large and drives the velocity toward zero, effectively suppressing fluid motion in the solid phase. This enthalpy-porosity-based damping formulation is widely used in phase-change simulations to mimic the momentum sink in the semi-solid region.

### 2.5. Initial and Boundary Conditions

In the numerical simulation, the initial temperatures of both the substrate and the powder were set to room temperature (approximately 293.15K), and the surrounding air domain was assigned the same temperature to represent thermal equilibrium prior to laser irradiation. The bottom of the substrate was in close contact with a cooling plate and was therefore approximated using a constant temperature boundary condition to reflect its strong heat dissipation effect. The lateral surfaces of the substrate and the free surface of the clad layer were considered for heat exchange with the environment. The boundary conditions at the air–contact surfaces were modeled using a combination of convection and thermal radiation, with a convective heat transfer coefficient of $25\;W/(m^{2}\cdot K)$ and a surface emissivity of 0.3. The selected emissivity corresponds to a relatively smooth metallic surface condition and is commonly adopted in laser processing simulations to provide a consistent estimation of radiative heat loss. To simplify the model and avoid introducing additional uncertainty associated with temperature-dependent emissivity, a constant value is employed. To simplify the model, symmetry was exploited, and the symmetry planes were treated as adiabatic boundaries to ensure energy conservation within the computational domain.

## 3. Results

### 3.1. Microstructural Evolution Under Different Scanning Speeds

#### 3.1.1. Dendritic Structure Analysis

**Figure 2:**
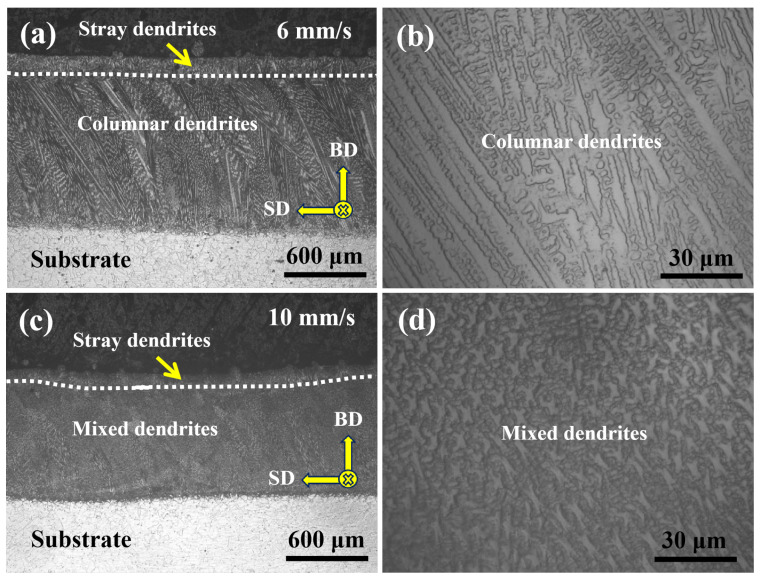
Optical microscopy (OM) images of specimens fabricated at different laser scanning speeds: (**a**) longitudinal section morphology of the clad layer at P600/V6; (**b**) P600/V6 microstructure; (**c**) longitudinal section morphology of the clad layer at P600/V10; (**d**) P600/V10 microstructure. BD: build direction; SD: scanning direction.

Figure 2 presents the optical microscopy (OM) morphologies of specimens fabricated by CW-LAM under different laser scanning speeds. Figure 2a,b correspond to the P600/V6 specimens, while Figure 2c,d correspond to the P600/V10 specimens. At the macroscopic level, all specimens exhibit a distinct single deposited layer and its corresponding dendritic microstructure. Due to fluctuations of the melt pool during fabrication, the top surface of the deposited layer displays slight undulations. At a lower scanning speed (P600/V6, Figure 2a), the deposited layer surface appears relatively smooth, indicating that the higher energy input per unit length facilitates better melt pool spreading and self-leveling. In contrast, at a higher scanning speed (P600/V10, Figure 2c), the deposited layer surface shows increased undulations and denser melt pool traces, reflecting reduced energy input and a shorter melt pool lifetime, which compromises melt pool stability. At the microstructural level, the P600/V6 specimen (Figure 2b) primarily consists of columnar dendrite bundles that grow epitaxially along the build direction (BD) from the substrate toward the top of the deposited layer, with growth trajectories deviating only slightly from the scanning direction (SD), demonstrating a pronounced preferential orientation. The primary dendrite arm spacing (PDAS) was measured to be approximately 7.9 μm, indicating a solidification process characterized by a high temperature gradient and a relatively low solidification rate, corresponding to a high ratio of temperature gradient to solidification rate (G/R), which favors the stable growth of columnar grains. It should be noted that PDAS is primarily governed by the cooling rate (G×R), which is generally inversely proportional to dendrite arm spacing. In the present condition, the relatively larger PDAS reflects a moderate cooling rate, while the concurrently high G/R contributes to the stability of directional columnar dendrite growth. Therefore, the observed microstructure results from the combined effect of G×R and G/R, rather than a single solidification parameter.

In contrast, the P600/V10 specimen (Figure 2d) exhibits a mixed dendritic microstructure consisting of both columnar and equiaxed dendrites. The overall preferential orientation is significantly weakened, although locally quasi-parallel alignment can still be observed. The primary dendrite arm spacing (PDAS) is refined to approximately 5.6μm. This indicates that, with increasing scanning speed, the energy input per unit length decreases while the melt pool cooling rate increases, thereby enhancing local constitutional undercooling and reducing the stability of columnar dendrite growth. As a result, new heterogeneous nucleation events are promoted near the solid–liquid interface. Consequently, the solidification conditions gradually evolve from those favoring columnar growth toward a columnar-to-equiaxed transition (CET). Overall, under a constant laser power of 600W, increasing the scanning speed reduces the linear energy input and increases the cooling rate, thereby promoting the transformation of the microstructure from coarse, directionally oriented columnar dendrites to refined mixed dendrites within the deposited layer. This behavior can be further understood from the perspective of solidification stability. An increased cooling rate (G×R) enhances constitutional undercooling at the solid–liquid interface, which promotes heterogeneous nucleation ahead of the advancing interface. Meanwhile, the simultaneous decrease in G/R reduces the morphological stability of columnar growth. The combined effect facilitates the columnar-to-equiaxed transition (CET), resulting in the formation of refined mixed dendritic structures.

**Figure 3:**
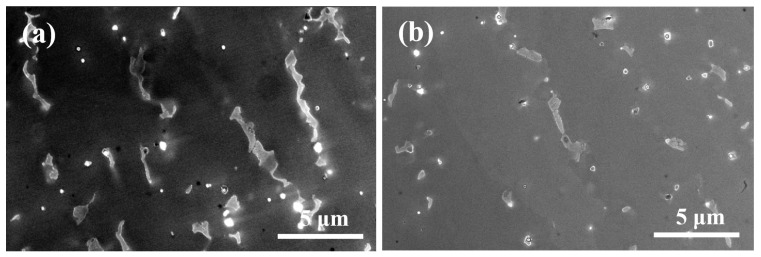
Representative scanning electron microscopy (SEM) morphologies of specimens fabricated under different laser scanning speeds: (**a**) SEM morphology of the specimen fabricated at a scanning speed of 6mm/s; (**b**) SEM morphology of the specimen fabricated at a scanning speed of 10mm/s.

Figure 3 presents representative scanning electron microscopy (SEM) morphologies of specimens fabricated under a constant laser power of 600W at different scanning speeds. Overall, a certain number of secondary phase particles can be observed in the interdendritic regions under all parameter conditions, indicating pronounced segregation and precipitation of solute elements such as Nb during the final stages of solidification. However, the spatial distribution and continuity of these particles evolve systematically with scanning speed. At a lower scanning speed (P600/V6, Figure 3a), the interdendritic particles exhibit distinct linear enrichment, with most particles aligned along the interdendritic channels and interconnected to form continuous chain-like networks. This suggests that the melt pool cooling rate is relatively low and the dendrite growth duration is sufficient, allowing solute elements ample time to diffuse and accumulate in the interdendritic regions, thereby promoting continuous particle precipitation and growth. When the scanning speed is increased to P600/V10 (Figure 3b), the chain-like networks show pronounced discontinuities, the connectivity between particles is weakened, and both particle size and volume fraction are reduced. This indicates that with increasing scanning speed, the melt pool solidification time decreases while the cooling rate increases, thereby limiting the redistribution time of interdendritic solutes. Consequently, particle coarsening is suppressed, and the continuous network gradually transforms into a discontinuous morphology.

Overall, with further increases in scanning speed, the interdendritic secondary phase particles gradually transform from continuous, chain-like networks into dispersed, isolated distributions. The particle size is further refined, and the spatial distribution becomes more uniform. This indicates that increasing the scanning speed fundamentally regulates the spatial distribution and morphological characteristics of interdendritic secondary phases by enhancing the cooling rate, shortening the solute segregation time, and promoting dendrite refinement.

#### 3.1.2. Elemental Analysis of Specimens

**Figure 4:**
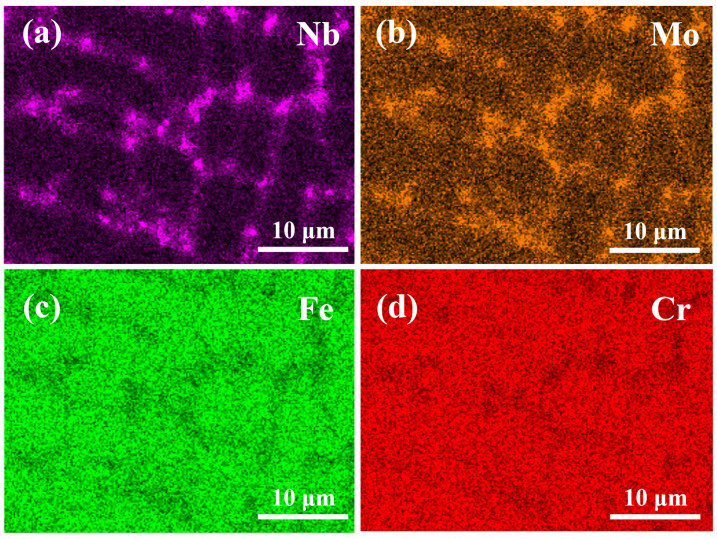
Representative SEM–EDS elemental maps of the fabricated specimens: (**a**) distribution of Nb; (**b**) distribution of Mo; (**c**) distribution of Fe; (**d**) distribution of Cr.

As shown in Figure 4, the SEM–EDS elemental maps of representative regions of the fabricated specimens are presented, where Figure 4a–d correspond to the distributions of Nb, Mo, Fe, and Cr, respectively. By correlating these maps with the spatial positions of interdendritic particles observed in the SEM morphology, pronounced elemental segregation can be identified. The signals of Nb and Mo (Figure 4a,b) are significantly enhanced in the interdendritic regions and closely coincide with the positions of the previously described particles, indicating evident enrichment. In contrast, Fe and Cr (Figure 4c,d) exhibit relatively weaker signals in the same regions and are more uniformly distributed within the dendritic matrix, suggesting clear depletion in these particle-rich areas. This elemental distribution pattern corroborates the interdendritic secondary-phase precipitation behavior observed under different scanning speeds. During the final stages of solidification, solute redistribution and dendritic segregation cause strongly segregating elements such as Nb and Mo to preferentially accumulate in the interdendritic liquid, forming Nb- and Mo-rich intermetallic particles during subsequent rapid solidification. Based on their chain-to-discrete evolution in the SEM images and their characteristic enrichment of Nb/Mo together with depletion of Fe/Cr, these particles located in the interdendritic regions can be identified as Laves phases [20]. Furthermore, with increasing scanning speed and the corresponding increase in cooling rate, the previously described particles gradually transform from continuous chain-like networks into fine and dispersed states. From a solidification perspective, the higher cooling rate enhances compositional undercooling and promotes nucleation, while simultaneously limiting the long-range diffusion of solute elements and particle growth. As a result, the continuous growth of Laves phases is suppressed, favoring their refined and discrete distribution.

Based on the combined analysis of OM, SEM, and EDS results, it can be concluded that under CW-LAM processing with a constant laser power of 600W, the scanning speed exerts a synergistic influence on the solidification microstructure and interdendritic precipitation behavior. With increasing scanning speed, the melt pool cooling rate is significantly enhanced, the dendrite size is progressively refined, and the redistribution time of interdendritic solutes is reduced. Consequently, the enrichment of strongly segregating elements such as Nb and Mo is weakened, leading to the fragmentation of the previously continuous chain-like Laves phases in the interdendritic regions into fine dispersed particles. Furthermore, EDS analysis indicates that the Nb/Mo segregation within the interdendritic regions decreases with increasing scanning speed, which is consistent with the observed microstructural refinement trend. These results demonstrate that, under CW-LAM processing conditions, increasing the scanning speed effectively suppresses solute segregation during solidification and reduces the continuity of Laves phase formation, thereby promoting a more homogeneous microstructure and improving the metallurgical quality of the deposited layer.

### 3.2. Numerical Simulation of Melt Pool Temperature Field in Continuous-Wave Laser Additive Manufacturing

#### 3.2.1. Evolution Characteristics of the Melt Pool Temperature Field Under Continuous-Wave Laser Irradiation

**Figure 5:**
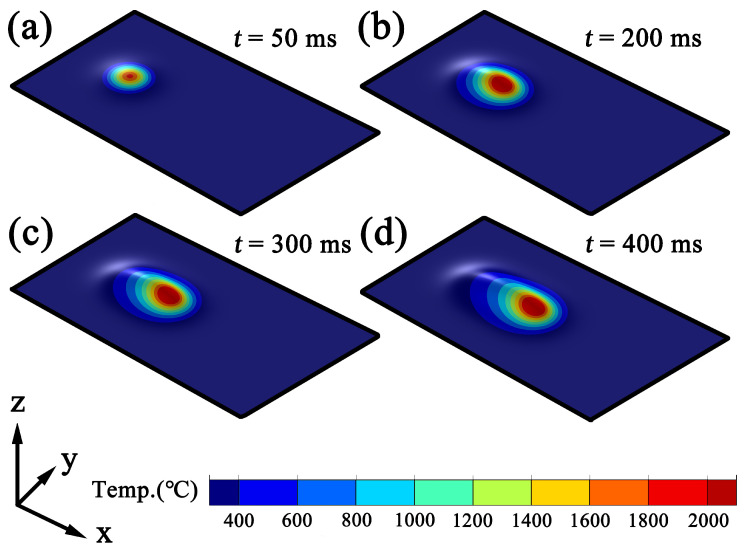
Melt pool formation at a scanning speed of 6mm/s: (**a**) t=50ms; (**b**) t=200ms; (**c**) t=300ms; (**d**) t=400ms.

**Figure 6:**
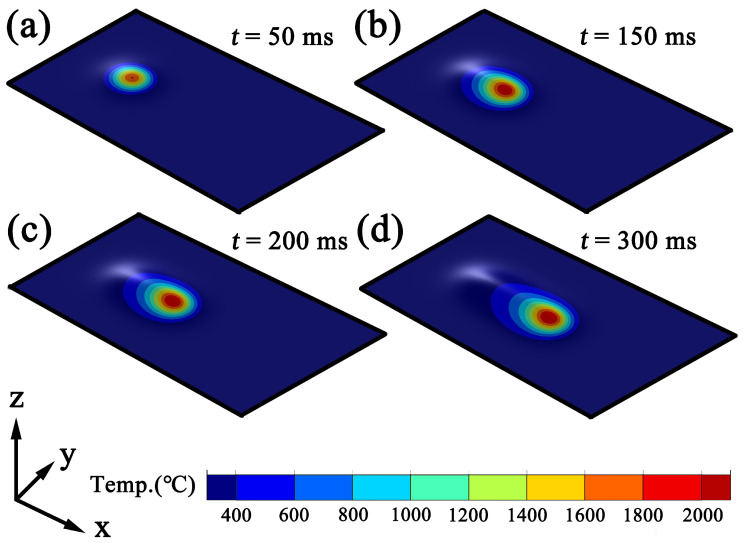
Melt pool formation at a scanning speed of 10mm/s: (**a**) t=50ms; (**b**) t=150ms; (**c**) t=200ms; (**d**) t=300ms.

Figure 5 and Figure 6 present the numerically simulated temporal evolution of the temperature field during the formation of the clad layer at scanning speeds of 6mm/s and 10mm/s, respectively. Both cases exhibit the typical motion characteristics of a laser cladding heat source, in which the high-temperature region elongates along the scanning direction and forms a stable ellipsoidal melt pool. However, the evolution rate of the melt pool and the time required to reach its maximum configuration differ significantly between the two scanning speeds. At a scanning speed of 6mm/s (Figure 5a–d), the melt pool exhibits an approximately circular high-temperature zone at the initial stage (t=50ms), indicating that the laser energy is initially accumulated in a localized region. As the time increases to t=200ms, the high-temperature zone begins to elongate along the scanning direction, and the melt pool gradually develops into an ellipsoidal structure with a defined aspect ratio. When the time further increases to t=300ms, the melt pool length continues to grow, and a temperature gradient along the scanning direction is progressively established, exhibiting a characteristic tailing profile. Finally, at approximately t=400ms, the melt pool morphology stabilizes and the high-temperature region reaches its maximum size, indicating that a quasi-steady-state melt pool has essentially formed. Therefore, at a scanning speed of 6mm/s, the melt pool attains its maximum configuration at approximately 400ms.

In contrast, at a scanning speed of 10mm/s (Figure 6a–d), the melt pool evolves significantly faster. At t=50ms, the high-temperature zone initially appears approximately circular; however, due to the higher scanning speed, the heat source travels a greater distance and the melt pool rapidly elongates along the scanning direction. By t=150ms, a distinct ellipsoidal high-temperature region is formed, and by t=200ms its spatial dimensions have essentially reached a stable configuration. At t=300ms, further changes in melt pool morphology become negligible, indicating that the system has entered a stable thermal equilibrium stage. Therefore, under the 10mm/s condition, the melt pool reaches its maximum quasi-steady-state configuration at approximately 200–300ms, which is notably earlier than that observed under the lower scanning speed.

Overall, as the scanning speed increases from 6mm/s to 10mm/s, the time required for the melt pool to reach a stable configuration is significantly reduced, indicating that higher scanning speeds shorten the local heat accumulation time and accelerate the melt pool formation process. Additionally, under higher scanning-speed conditions, the elongation of the melt pool along the scanning direction becomes more pronounced, while the overall area of the high-temperature region becomes relatively smaller, reflecting the characteristics of reduced energy input per unit length and an increased cooling rate. With further extension of the irradiation time, both the temperature and the dimensions of the melt pool remain essentially unchanged, indicating that the melt pool has fully reached a stable state. Under continuous-wave laser conditions, the melt pool temperature distribution and morphological dimensions remain stable over time, exhibiting no significant variations. This stable stage provides a reliable basis for the subsequent extraction of solidification parameters (*G* and *R*), as it ensures that the calculated values are not influenced by transient melt pool evolution or incomplete clad layer formation. Therefore, all solidification parameters presented in the following analysis are obtained after the melt pool has reached this quasi-steady state.

#### 3.2.2. Melt Pool Thermal Behavior Under Typical CW-LAM Processing

**Figure 7:**
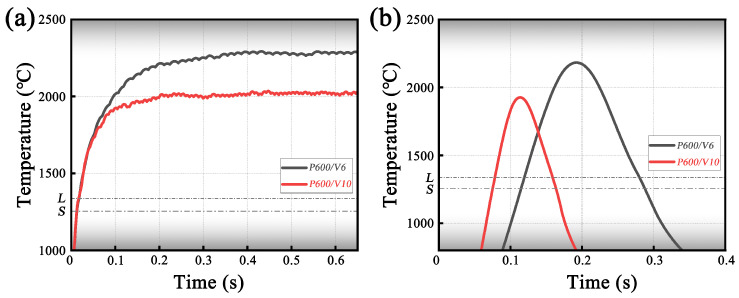
Peak melt pool temperature and temperature at a fixed point under different scanning speeds: (**a**) temporal variation of the peak melt pool temperature; (**b**) temperature evolution at a fixed position (x=1.5mm).

Figure 7 illustrates the temperature evolution characteristics of the melt pool under a laser power of 600W at two scanning speeds, 6mm/s (P600/V6) and 10mm/s (P600/V10). Figure 7a shows the temporal variation of the peak melt pool temperature, while Figure 7b presents the thermal cycling curves at a fixed position of x=1.5mm. Together, these results highlight the significant differences in melt pool thermal input and thermal cycling behavior caused by changes in scanning speed.

As shown in Figure 7a, the peak melt pool temperature rapidly increases during the initial stage of laser irradiation under both process conditions. However, the peak temperature under the lower scanning speed condition (P600/V6) is consistently higher than that under the higher scanning speed condition (P600/V10). Specifically, the peak temperature for P600/V6 stabilizes at approximately 2250–2300 °C, whereas for P600/V10 it stabilizes near 2000 °C. This observation indicates that, at lower scanning speeds, the energy input per unit length is higher and the laser dwells longer in the local region, allowing heat to accumulate continuously within the melt pool and resulting in a higher quasi-steady-state peak temperature. In contrast, increasing the scanning speed accelerates the movement of the heat source, shortens the local heat accumulation time, and consequently reduces the maximum melt pool temperature.

Figure 7b illustrates the thermal cycling characteristics at a fixed monitoring point under the two scanning-speed conditions. Both processes exhibit a typical “rapid heating–peak–rapid cooling” thermal cycle; however, the peak temperature, duration, and width of the thermal cycle differ markedly. For the P600/V6 process, the peak temperature reaches approximately 2100–2200 °C, and the high-temperature dwell time is relatively long, resulting in a thermal cycle width that is significantly larger than that of P600/V10. In contrast, under the P600/V10 condition, the peak temperature decreases to around 1900°C, and the curve becomes noticeably narrower, indicating a shorter high-temperature dwell time and a faster cooling rate of the melt pool. These results demonstrate that increasing the scanning speed substantially shortens the local thermal cycle period and enhances the solidification cooling rate of the material.

The above results indicate that under the 6mm/s condition, the melt pool requires a longer time (∼400ms) to reach its stable maximum configuration, forming a larger high-temperature region. Consequently, it exhibits a higher peak temperature and a wider thermal cycle. In contrast, under the 10mm/s condition, the melt pool attains a stable configuration within approximately 200–300ms, and the high-temperature region is comparatively smaller, resulting in a lower peak temperature and a more concentrated thermal cycle.

#### 3.2.3. Temperature Field Sampling and Morphological Validation

**Figure 8:**
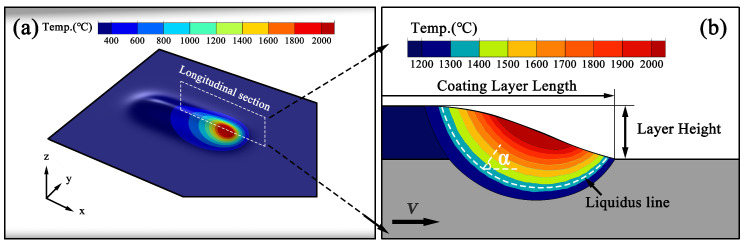
Simulated temperature field and geometric features of the clad layer in CW-LAM: (**a**) three-dimensional temperature field distribution of the melt pool during laser scanning; (**b**) longitudinal section sampling scheme and geometric parameters used for the extraction of solidification parameters.

Figure 8 illustrates the temperature field of the clad layer and the longitudinal-section sampling scheme used to extract solidification parameters at the melt pool boundary and to analyze the solidification behavior of the melt pool. Figure 8a presents the three-dimensional temperature distribution during laser scanning. The high-temperature region is concentrated near the laser interaction zone and gradually extends along the scanning direction, forming a characteristic trailing temperature field behind the laser. To clearly analyze the thermal conditions governing solidification, a longitudinal section along the scanning direction is selected within the melt pool, as shown in Figure 8a. This section allows a more intuitive observation of the temperature distribution and the morphology of the solid–liquid interface. Figure 8b shows the temperature contour distribution and geometric features of the selected longitudinal section. The boundary corresponding to the liquidus temperature (1336 °C) is defined as the liquidus line, which represents the solid–liquid interface used for extracting solidification parameters. Two geometric parameters are defined to describe the macroscopic morphology of the deposited layer. The Coating Layer Length represents the extension length of the clad layer along the scanning direction, while the Layer Height denotes the height of the deposited layer above the substrate. In order to systematically extract solidification parameters along the melt pool boundary, sampling points are selected along the liquidus line. At each sampling location, the angle α is defined as the angle between the horizontal direction and the normal line to the tangent of the liquidus line at that point. This definition provides a geometric reference for determining the local growth direction during solidification. The arrow *v* indicates the laser scanning direction and scanning velocity. Based on the thermal field along the longitudinal section, the temperature gradient *G* and the solidification rate *R* can be evaluated along the liquidus line. These parameters are then used to calculate the solidification criteria G/R and G×R, which play a key role in determining the columnar-to-equiaxed transition (CET) behavior of the solidified microstructure.

**Figure 9:**
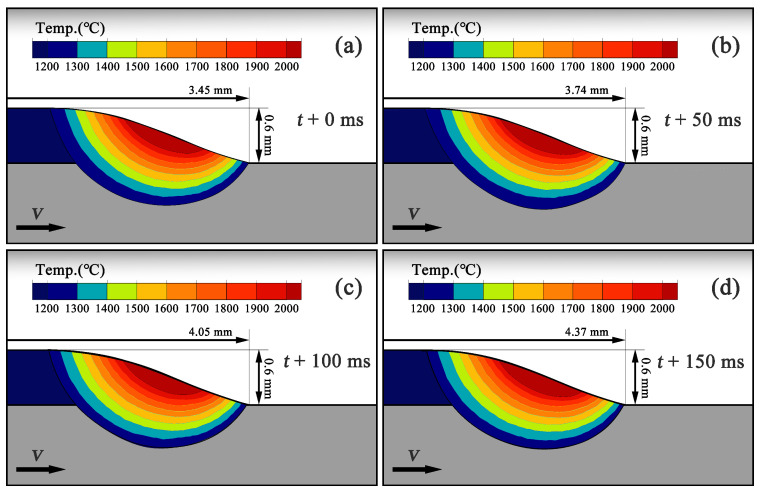
Temporal evolution of the melt pool cross-sectional morphology after reaching a stable configuration: (**a**) t+0ms; (**b**) t+50ms; (**c**) t+100ms; (**d**) t+150ms.

Figure 9 illustrates the temporal evolution of the temperature field along the longitudinal section of the melt pool after it has reached a stable configuration, starting from t+0ms. Figure 9a–d correspond to t+0ms, t+50ms, t+100ms, and t+150ms, respectively. Overall, within this time interval, the melt pool morphology, dimensions, and temperature distribution remain essentially stable, exhibiting only minor periodic fluctuations. The temperature contours within the melt pool maintain a consistent pattern, with the high-temperature region concentrated at the melt pool center and a stable temperature gradient established along the scanning direction. These observations indicate that under CW-LAM processing, once the melt pool is established, its temperature field and geometric configuration reach a quasi-steady-state solidification condition. In this state, the overall melt pool morphology remains relatively stable as the laser scans, with only slight periodic variations.

This quasi-steady-state characteristic indicates that during the stable stage of the melt pool, the solidification behavior at the melt pool boundary is primarily determined by spatial position, with minimal influence from temporal variations. The temperature gradient *G* and the solidification rate *R* at different locations along the solid–liquid interface depend mainly on their relative positions within the melt pool rather than the specific instantaneous time. Therefore, when analyzing melt pool solidification parameters, emphasis can be placed on the solidification behavior at different positions along the rear edge of the melt pool. Based on this consideration, five representative points were selected along the solid–liquid interface from the top to the bottom of the longitudinal section at the rear of the melt pool in the simulation results. The temperature gradient *G* and the solidification rate *R* were extracted at each point to analyze the temporal variation of the solidification parameters G/R and G×R. Since the melt pool is overall in a quasi-steady state, these parameters exhibit only minor fluctuations over time, allowing them to reliably reflect the local solidification conditions at different spatial positions. This approach provides a theoretical basis for further interpreting the observed dendrite growth patterns and microstructural evolution in the experiments.

#### 3.2.4. Melt Pool Morphology Under Different Scanning Speeds at the Same Time

**Figure 10:**
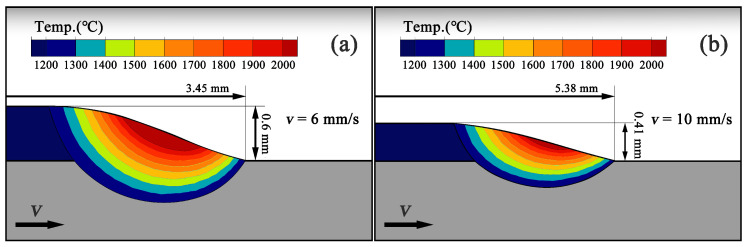
Schematic of melt pool cross-sectional morphologies under two scanning speeds: (**a**) v=6mm/s; (**b**) v=10mm/s.

As shown in Figure 10, the longitudinal section temperature fields and melt pool morphologies at t=0.5s are compared under two scanning speed conditions, where Figure 10a corresponds to v=6mm/s and Figure 10b corresponds to v=10mm/s. It is evident that variations in scanning speed significantly affect both the melt pool geometry and the temperature field distribution. Under the lower scanning speed condition (v=6mm/s, Figure 10a), the melt pool exhibits a relatively deep and tall profile, with a clad layer length of approximately 3.45mm and a layer height of about 0.6mm. The high-temperature region is extensive, the central melt pool temperature is elevated, and the isotherms extend noticeably into the substrate, resulting in a pronounced penetration depth. This indicates that at lower scanning speeds the laser dwells longer in the local region and the energy input per unit length is higher, leading to significant heat accumulation within the melt pool, a higher peak temperature, and a larger melt pool volume. In contrast, under the higher scanning speed condition (v=10mm/s, Figure 10b), the melt pool morphology changes markedly. The clad layer length increases to approximately 5.38mm, while the layer height decreases to about 0.41mm, resulting in a flatter melt pool profile with reduced penetration depth. The temperature contour distribution shows a smaller high-temperature region and a lower central melt pool temperature, indicating that with increased scanning speed the laser dwell time is reduced, local heat accumulation is weakened, and the overall melt pool temperature decreases.

It can be observed that under higher scanning speed conditions, the melt pool boundary exhibits a relatively straighter contour, and the temperature gradient along the substrate direction becomes more concentrated. This indicates that heat is primarily conducted and dissipated along the substrate, resulting in a stronger unidirectional heat dissipation characteristic and an increased solidification cooling rate. Such a thermal conduction feature is conducive to establishing a stable temperature gradient, which provides favorable conditions for directional crystal growth along the heat flow. Overall, as the scanning speed increases from 6mm/s to 10mm/s, the clad layer morphology exhibits a reduced layer height, a shallower penetration depth, a lower peak temperature, and a more flattened melt pool, accompanied by a reduced high-temperature region.

## 4. Discussion

### 4.1. Characteristics of Solidification Parameters at the Melt Pool Boundary

**Figure 11:**
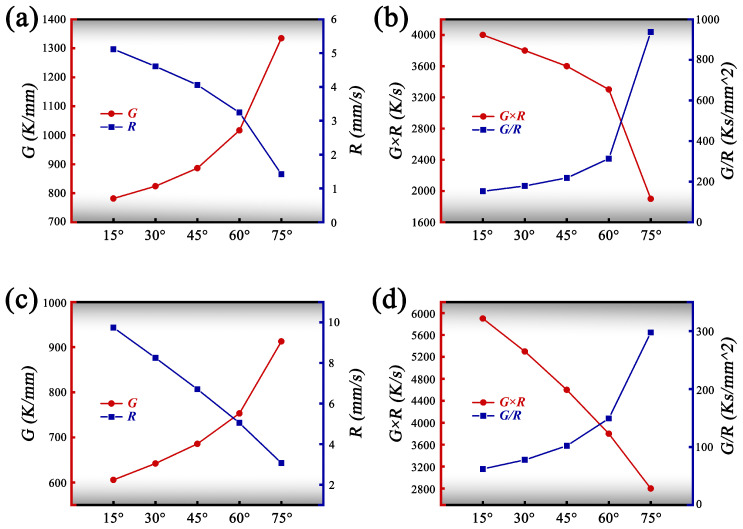
Solidification parameters at the melt pool solid–liquid interface under different scanning speeds: (**a**,**b**) v=6mm/s; (**c**,**d**) v=10mm/s.

Figure 11 presents the solidification parameters along the melt pool cross-section under two scanning speed conditions. Figure 11a,b show the variation of solidification parameters at different positions (15–$75^{\circ }$) along the solid–liquid interface under the v=6mm/s condition, while Figure 11c,d correspond to v=10mm/s. Overall, as the sampling points move from the upper to the lower region of the melt pool, the temperature gradient *G* and the solidification rate *R* exhibit opposite trends. Consequently, the derived combined parameters—the cooling rate G×R and the morphological criterion G/R—also display distinct corresponding variations.

Under the v=6mm/s condition (Figure 11a), the temperature gradient *G* gradually decreases from approximately 1300K/mm to around 900K/mm along the solid–liquid interface. Meanwhile, the solidification rate *R* continuously increases from about 0.8mm/s to approximately 5mm/s. Consequently, the cooling rate G×R (Figure 11b) exhibits an overall decreasing trend, whereas the morphological criterion G/R continuously increases. A higher G/R value indicates enhanced stability of the solid–liquid interface, favoring sustained epitaxial growth along the temperature gradient and promoting the formation and directional extension of columnar dendrites. At the same time, the relatively low cooling rate (G×R) prolongs the residence time of the liquid phase during the final stage of solidification, providing sufficient conditions for solute redistribution and accumulation in the interdendritic regions, thereby enhancing elemental segregation between dendrites.

Under the v=10mm/s condition (Figure 11c), the temperature gradient *G* similarly decreases along the solid–liquid interface, from approximately 950K/mm to 650K/mm, while the solidification rate *R* increases from around 2.5mm/s to nearly 9mm/s. In Figure 11d, although the cooling rate G×R gradually decreases along the interface, its overall level is noticeably higher than that under the 6mm/s condition. Meanwhile, the G/R values are generally lower compared with those at the lower scanning speed. The higher G×R indicates an increased cooling rate during solidification, which shortens the crystal growth time and promotes dendrite refinement. Conversely, the lower G/R reduces the morphological stability of the solid–liquid interface, leading to more dispersed growth orientations, increased dendrite branching, and a greater tendency for the columnar-to-equiaxed transition (CET). Additionally, the combination of higher cooling rates and faster interface advancement shortens the time available for solute diffusion and accumulation in the interdendritic liquid, thereby weakening the sustained enrichment of solute elements and reducing interdendritic segregation.

Therefore, the synergistic variation of the solidification parameters *G*, *R*, G×R, and G/R directly governs the crystal growth mode and solute redistribution behavior. A higher G/R favors the maintenance of stable columnar dendrite growth, while a higher G×R corresponds to faster cooling conditions and finer dendrite scales, simultaneously suppressing interdendritic solute segregation.

### 4.2. Integrated Mechanism of Scanning Speed on Solidification Kinetics and Laves Phase Evolution

By integrating the melt pool thermal evolution, solidification parameter distribution, and microstructural characterization, it is evident that scanning speed systematically governs melt pool solidification behavior, crystal growth modes, and Laves phase formation during CW-LAM. As the scanning speed increases, the thermal input decreases, leading to a narrower high-temperature region, a flatter melt pool morphology, reduced heat accumulation, and an increased cooling rate.

At the solidification kinetics level, this behavior is reflected by an increase in the solidification rate *R* and a relative decrease in the temperature gradient *G*, resulting in a higher cooling rate (G×R) and a lower morphological stability parameter (G/R). This combination directly regulates both dendrite scale and growth mode.

Under low scanning speed conditions, the relatively high G/R maintains directional solidification stability, promoting epitaxial growth of columnar dendrites. Meanwhile, the lower cooling rate (G×R) prolongs the solidification process, creating a wider kinetic time window for solute redistribution. This facilitates the accumulation of Nb and Mo in interdendritic regions, leading to the formation of continuous chain-like Laves phases.

In contrast, higher scanning speeds increase G×R and reduce G/R, resulting in dendrite refinement and reduced interfacial stability, which promotes the transition from columnar to mixed columnar–equiaxed structures. At the same time, the significantly compressed kinetic time window limits solute diffusion and accumulation, thereby weakening elemental segregation and transforming Laves phases into fine and dispersed particles.

Overall, scanning speed regulates melt pool thermal history and solidification kinetics, thereby coupling dendrite growth behavior with solute redistribution. Lower scanning speeds favor columnar growth but promote Laves phase formation, whereas higher scanning speeds refine the microstructure and effectively suppress deleterious phase precipitation.

## 5. Conclusions

This study systematically investigated the solidification behavior of the melt pool and the evolution of microstructure under different scanning speeds during CW-LAM by combining experimental characterization with high-fidelity multiphysics numerical simulations. By quantitatively analyzing the key solidification parameters along the entire liquidus boundary of the melt pool, including the temperature gradient *G* and the solid–liquid solidification rate *R*, the fundamental mechanism by which scanning speed regulates melt pool solidification kinetics and Laves phase formation was revealed. The main conclusions are summarized as follows:

(1) Scanning speed significantly influences the thermal behavior and solidification environment of the melt pool. With increasing scanning speed, both the peak temperature of the melt pool and the extent of the high-temperature region decrease markedly, resulting in reduced heat accumulation. Meanwhile, the cooling rate of the melt pool increases substantially, thereby accelerating the non-equilibrium solidification process and altering the local solidification environment.
(2) A unified solidification control mechanism based on *G* and *R* is revealed. Increasing the scanning speed leads to an increase in the solidification rate *R* and a relative decrease in the temperature gradient *G*, resulting in an elevated cooling rate (G×R) and a reduced morphological stability parameter (G/R). This synergistic variation governs both dendrite scale and growth mode, where higher G×R promotes dendrite refinement, while lower G/R reduces interfacial stability and facilitates the transition from stable columnar dendrites to a mixed columnar–equiaxed structure.
(3) The formation mechanism of Laves phases is interpreted from a kinetic perspective through the combined use of high-fidelity numerical simulation and experimental characterization, which provide complementary validation of both thermal behavior and microstructural evolution. Under low scanning speed conditions, the relatively low cooling rate and prolonged solidification time create a relatively wide kinetic time window for solute redistribution, enabling sufficient diffusion and accumulation of solute elements in interdendritic regions, and thus leading to the formation of continuous chain-like Laves phases. In contrast, increasing the scanning speed enhances the cooling rate and significantly compresses this kinetic time window, thereby limiting the time available for solute redistribution, suppressing elemental segregation, and transforming the Laves phase morphology into fine and dispersed particles. This result highlights that the evolution of deleterious phases in laser additive manufacturing can be effectively controlled by regulating the kinetic time window associated with non-equilibrium solidification.

## Figures and Tables

**Table 1:** Chemical composition of the powder (mass fraction %).

Cr	Fe	Nb	Mo	Ti	Al	Ni
19.00	18.50	5.20	2.90	0.75	0.60	Bal.

## Data Availability

The original contributions presented in this study are included in the article. Further inquiries can be directed to the corresponding author.

## Abbreviations

The following abbreviations are used in this manuscript:

PDAS	Primary Dendrite Arm Spacing
OM	Optical Microscopy
CET	Columnar-to-equiaxed Transition
SEM	Scanning Electron Microscopy
EDS	Energy Dispersive X-ray Spectroscopy
